# Epitranscriptomics of Mammalian Mitochondrial Ribosomal RNA

**DOI:** 10.3390/cells9102181

**Published:** 2020-09-27

**Authors:** Ivan Laptev, Olga Dontsova, Petr Sergiev

**Affiliations:** 1 Belozersky Institute of Physico-Chemical Biology, Lomonosov Moscow State University, 119992 Moscow, Russia; whiteswan92@gmail.com (I.L.); olga.a.dontsova@gmail.com (O.D.); 2 Center of Life Sciences, Skolkovo Institute of Science and Technology, Skolkovo, 143028 Moscow Region, Russia; 3 Department of Chemistry, Lomonosov Moscow State University, 119992 Moscow, Russia; 4 Shemyakin-Ovchinnikov Institute of Bioorganic Chemistry, 117997 Moscow, Russia; 5 Institute of Functional Genomics, Lomonosov Moscow State University, 119992 Moscow, Russia

**Keywords:** mitochondria, ribosome, translation, RNA modification, ribosome assembly

## Abstract

Modified nucleotides are present in all ribosomal RNA molecules. Mitochondrial ribosomes are unique to have a set of methylated residues that includes universally conserved ones, those that could be found either in bacterial or in archaeal/eukaryotic cytosolic ribosomes and those that are present exclusively in mitochondria. A single pseudouridine within the mt-rRNA is located in the peptidyltransferase center at a position similar to that in bacteria. After recent completion of the list of enzymes responsible for the modification of mammalian mitochondrial rRNA it became possible to summarize an evolutionary history, functional role of mt-rRNA modification enzymes and an interplay of the mt-rRNA modification and mitoribosome assembly process, which is a goal of this review.

## 1. Introduction

While epigenetics has been long known as an essential mechanism for gene expression control, epitranscriptomics, a field of RNA modification, has rather recently come into focus of the scientific community. Ribosomal RNA in the entire range of species contains modified, most frequently methylated residues and pseudouridines [1]. Ribosomal RNA is functionalized by non-standard nucleotides predominantly located in the functional centers of the ribosome [2,3,4]. These nucleotides demonstrate variable degrees of conservation, from the universal to relatively narrow. Among different types of ribosomes, mitochondrial ribosomes are only minimally modified. Mammalian mitochondrial rRNAs contain nine methylated residues mapped for hamster mt-rRNAs in the early 1980s [5,6] and one pseudouridine identified in 1997 [7]. Still, up to 2012 [8], only a single gene coding for the mammalian mitochondrial rRNA modification enzyme was known. Only recently, in 2019–2020 the list of mammalian mt-rRNA modification enzymes has been completed [9,10,11,12,13], allowing us to summarize the entire inventory of mammalian mt-RNA modification machinery (Table 1), which is the subject of current review and several other excellent reviews on this topic [14,15,16].

Mitochondria is a power factory of eukaryotic cells, also responsible for apoptosis, synthesis of a number of key metabolites and many other functions [17]. According to the hypothesis of endosymbiosis, mitochondria evolved from eubacterial symbiont closely related to α-proteobacteria [18]. In the course of eukaryote evolution the predominant majority of its genes migrated into the host nucleus [19]. Mitochondria have a small circular genome and machineries for transcription and translation. To fulfill its main function, an ATP production through oxidative phosphorylation, mitochondria possesses five multisubunit protein complexes in its inner membrane. Electron transfer chain (ETC) complexes I to IV are building up transmembrane potential fueled by oxygen reduction with NADH and succinate, while ATP-synthase consumes the potential formed by ETC and synthesizes ATP. In addition to the nuclear encoded components, complexes of the oxidative phosphorylation contain, in a case of mammals, 13 proteins, encoded in the mitochondrial genome, such as 7 subunits of NADH-dehydrogenase (complex I), cytochrome b (complex III), 3 subunits of cytochrome c oxidase (complex IV) and 2 subunits of ATP-synthase (complex V). All other proteins needed for proper maintenance and function of mitochondria are encoded in the nucleus including, remarkably, all subunits of the succinate dehydrogenase (complex II), whose activity is thus being a useful control independent of the mitochondrial gene expression machinery. In addition to the protein-coding genes, mammalian mitochondrial genome codes for the 12S and 16S rRNAs, components of the small and large subunits of mitoribosome, and 22 tRNAs. All these genes are transcribed from three promotors, HSP1, HSP2 (heavy strand promoters) and LSP (light strand promoter). The product started with HSP1 promoter is limited to 12S and 16S rRNA species and two tRNAs–tRNA^Phe^ and tRNA^Val^, while transcript started with HSP2 promoter extends further to cover almost the entire genome [20,21]. The transcript started with LSP promoter corresponds to the opposite DNA strand relative to that of HSP1 and HSP2 driven transcripts. It spans almost the entire genome, excluding the regulatory region and an antisense to the rRNA coding part.

## 2. Peculiar Features of Mitochondrial Translation Apparatus

**Figure 1:**
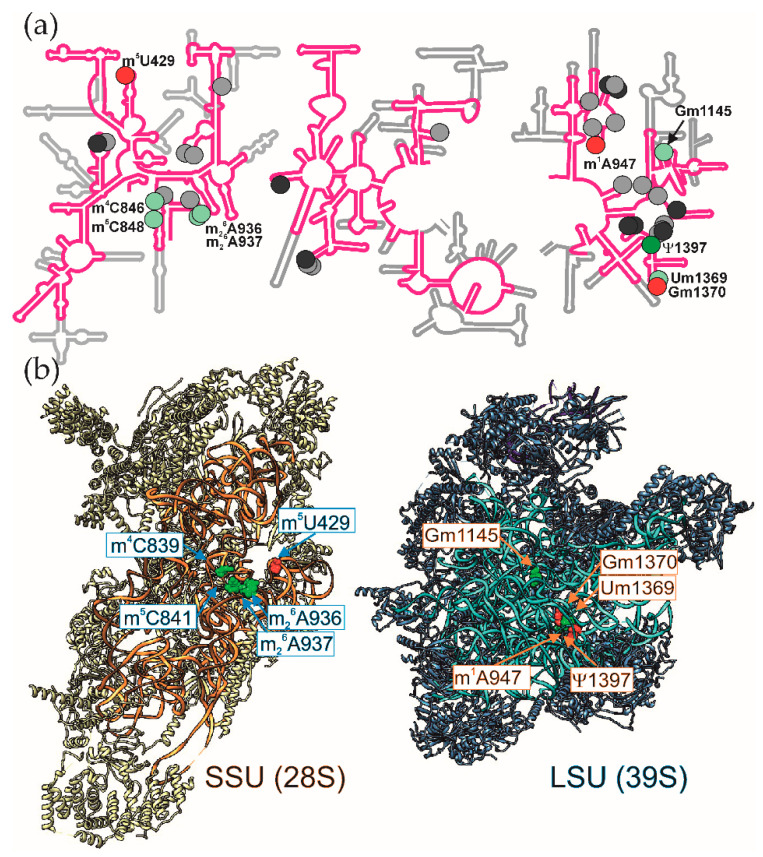
Location of the modified nucleotides in the mammalian mitochondrial rRNA. (**a**) Secondary structure [34] of human mitochondrial 12S rRNA (left) and 16S rRNA (right) are shown in pink superimposed onto that of *E. coli* 16S rRNA and 23S rRNA shown in grey. Nucleotides methylated in both bacterial and mitochondrial rRNA are shown by light green circles, while conserved pseudouridine is shown as a dark green circle. Red circles correspond to nucleotides modified exclusively in mt-rRNA. Modified nucleotides present in *E. coli* rRNA, but not in mt-rRNA are shown by light grey (methylated) and dark grey (pseudouridinylated) circles. (**b**) Tertiary structure of the human mitochondrial small (left) and large (right) ribosomal subunits [35]. Ribosomal proteins of the small subunit are shown as yellow ribbon, while that of the large subunit as steel blue ribbon. 12S rRNA is shown as orange ribbon; 16S rRNA as cyan ribbon, tRNA of the large subunit as blue ribbon. Methylated nucleotides of the mt-rRNA are shown as spacefilled objects and marked by the same colors as on the panel (**a**). UCSF Chimera [36] was used to create this illustration.

Although mitochondrial ribosomes evolved most likely from bacterial ones, they differ from each other in composition [22], 3D structure [23] and properties of proteins they synthesize. While sedimentation coefficient of mitoribosome (55S) and its constituent subunits (28S and 39S) is somewhat lower than that of bacterial ones, the most striking difference resides in their composition. Mitoribosomes gradually lost various parts of their rRNA molecules (Figure 1a), which were commonly, but not necessarily, replaced with proteinaceous structures. Mitochondrial ribosomes acquired both entirely new r-proteins and mitochondrial specific extensions [24,25,26,27,28] of r-proteins that are conserved in other types of ribosomes. Spectacular changes were observed for the central protuberance of the large ribosomal subunit, where 5S rRNA was either lost, as in yeast [29,30], or replaced by mt-tRNA^Val^ or mt-tRNA^Phe^ [25,31] in mammalian mitoribosomes. Presence of specific proteins surrounding the peptide exit site of mitoribosomes, such as mL45 [25,32] might be explained by the specialization in the synthesis of highly hydrophobic transmembrane proteins [33].

Specific changes were observed for the mRNA binding channel. Anti-Shine-Dalgarno (aSD) part of bacterial small subunit rRNA is absent in mitoribosomes, in concert with the peculiar features of mt-mRNA (see below). mS38 protein, which is lacking in *E. coli*, although present (as an orthologous bS22) in some bacteria which have many leaderless mRNAs, is involved in formation of the channel accommodating mRNA part 5′ to the P-site. In yeast mitochondria this protein was demonstrated to selectively enhance translation of specific mitochondrial mRNAs [37]. A protein mS37, which is also uniquely found in mitoribosomes also contributes to the accommodation of mRNA 5′-region [38]. The 3′ part of mRNA interacts with another mitochondria-specific protein, mS39, which is also involved in mRNA selection [39]. It interacts with large proteinaceous LRPPRC-SLIRP complex mediating mRNA recruitment [40]. Many protein factors of mitochondrial translation also contain specific extensions, likely to have separate functions related to mitochondrial translation [41,42,43,44]. Among them remarkably are translation initiation factors, such as mtIF2, which possesses an extension functionally replacing bacterial IF1 [45], whose ortholog is absent in mitochondria and mtIF3, whose N- and C-terminal extensions have unique functions in mitochondria [43,46].

Specific features of mitochondrial ribosome and translation factors might be a product of co-evolution with mitochondrial type mRNAs. Unlike the majority of bacterial mRNAs, mitochondrial ones lack SD sequence. Moreover, in mammals, and unlike yeasts, mRNAs entirely lack or have minute 5′-untranslated regions. At the same time, two of them are bicistronic with overlapped open reading frames [47]. Mammalian mitochondria utilize non-AUG codons, such as AUU and AUA for the initiation of translation. Even more bizarre is termination of protein synthesis in mitochondria. While UGA codon is reassigned to the tryptophan in mammalian mitochondria, AGA and AGG codons are avoided altogether. In two instances human mt-mRNA coding regions ends with those codons, so that translation is terminated via -1 frameshifting, placing UAG in the A-site [48]. In many cases, stop codons are formed by polyadenylation of primarily processed transcripts which, after excision of tRNAs, ends with U or UA nucleotides [47]. Polyadenylation itself might not necessarily start within a stop codon. Some mRNAs have 3′-untranslated regions preceding post-transcriptionally added polyA tail, while single ND6 mRNA is not polyadenylated at all [8,49].

In line of all the above, the set of mammalian mitochondrial rRNA modifications, pretty much like entire mitochondrial translation apparatus, is a potluck of universally conserved core, ladle of bacterial-type, a pinch of archaeal/eukaryotic cytosolic-type and a spoonful of mitochondrial unique specialties.

## 3. Universally Conserved Core Modifications

Out of nine methylated nucleotides of the mitochondrial rRNA (Table 1) four are present in all types of ribosomes, thus being the most conserved ones.

### 3.1. 12S rRNA m_2_^6^A936, m_2_^6^A937

Nucleotides m_2_^6^A936 and m_2_^6^A937 are located in the loop of helix 45 near 3′-end of the 12S rRNA (Figure 1). In bacteria equivalent nucleotides m_2_^6^A1518/9 are modified by RsmA/KsgA [50,51,52], an enzyme whose inactivation resulted in kasugamycin resistance [50,53]. Absence of these nucleotides methylation was not found to affect ability of the 30S subunit to associate with fMet-tRNA, initiation factors and 50S subunit in vitro [52]. Single mutations of A1518, A1519 or other nucleotides in the stem of helix 45 didn’t affect ribosome assembly in vitro as well, although some of them decreased the binding effectiveness of tRNA, initiation factors and the whole translation efficiency [54,55].

Later it was shown that RsmA binds 30S subunit and prevents binding of IF3 and the large subunit [56]. Overexpression of catalytically dead RsmA mutant decreased subunit association and appeared to be more toxic than the corresponding gene knockout. This result leads to an idea that RsmA acts as a quality control switch releasing the subunit after completion of late assembly events [57]. In concert with this idea, rsmA deletion excessively retarded cell growth at 25 °C, when assembly defects are exacerbated [57], and caused accumulation of the small subunit rRNA precursor [57,58]. Apart from RsmA role in ribosome assembly, the methylation itself is also essential for the proper rRNA folding. Without methylation, contact interface between the 16S rRNA helices 45 and 44 is distorted leading to the alteration in the mRNA binding region [59].

Cytosolic ribosomes of eukaryotes also contain a similar pair of dimethylated adenosines, introduced by homologous Dim1 (yeast) [60] or DIMT1L (mammals) [61] methyltransferases. Existing evidences suggest an essential role of these proteins in ribosome construction, not only as the methyltransferase, but also as an assembly factor, since catalytically inactive Dim1 mutant expression was able to partially complement Dim1 deficiency [62].

**Figure 2:**
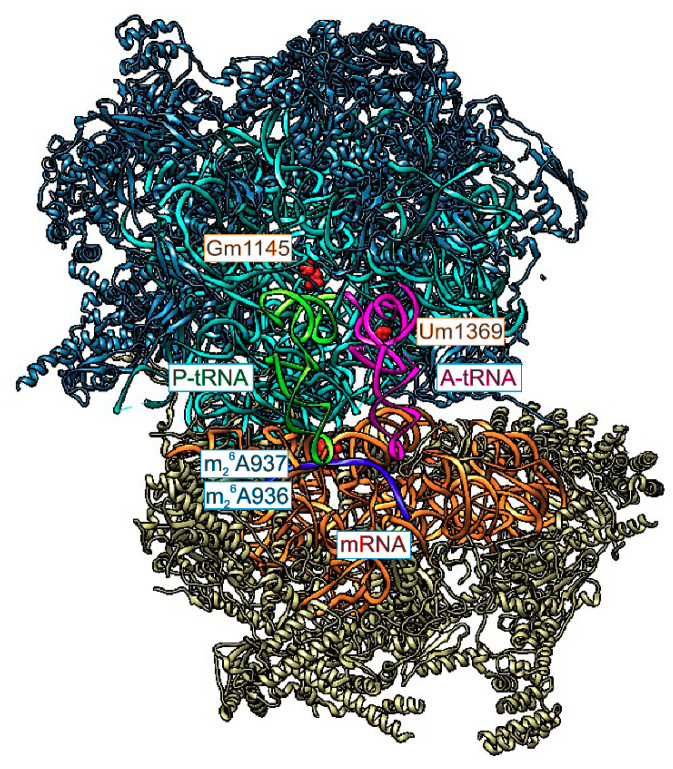
Location of the universally conserved methylated nucleotides, shown as red spacefilled objects and marked, in the mammalian mitochondrial rRNA relative to the ligands of the ribosome [69]. A slice through the top-viewed functional complex is shown. mRNA is shown as blue ribbon, A-site bound tRNA is shown as pink ribbon and P-site bound tRNA is shown as green ribbon. UCSF Chimera [36] was used to create this illustration.

In mammalian mitoribosomes nucleotides m_2_^6^A936 and m_2_^6^A937 (Figure 2) are modified by TFB1M [63] in a sequential order starting with A937 [64]. TFB1M and its paralogue, TFB2M, may also act as a transcription factor to activate transcription by POLRMT in vitro [65]. For transcription activation, TFB1M doesn’t need methyltransferase domain [66]. Later it was shown that most likely transcription activation is the main function of TFB2M, while major role of TFB1M is ribosome assembly and modification [67]. POLRMT interaction with TFB1M might be beneficial for the coordination between mitochondrial rRNA synthesis and modification [68].

Inactivation of Tfb1m in mice leads to embryonic lethality [70]. Tissue specific inactivation in heart and skeletal muscles results in cardiomyopathy. All such mice died before the age of 24 weeks. Mitochondrial mass in the heart of knockout mice was increased relative to that of the wild type mice, likely as a result of a compensatory mechanism. Absence of TFB1M led to a decreased stability of the small ribosomal subunit, faster 12S rRNA degradation and as a consequence, decreased amount of the 55S ribosomes. This might be indicative of m_2_^6^A936 and m_2_^6^A937 modification role in stabilization of the ribosomal small subunit structure in the conserved functional core region which is in contact with mRNA and the large ribosomal subunit (Figure 2).

Inactivation of Tfb1m gene caused a decreased amount of the small ribosomal subunits, diminished efficiency of mitochondrial translation [64], and decrease in the abundance of ETC complexes subunits, encoded in the mitochondrial genome [70]. Tfb1m inactivation in murine pancreatic β-cells also causes mitochondrial disfunction: such cells have decreased ETC complex activity, while the amount of mitochondria is elevated. As a consequence, such mice have decreased insulin secretion and diabetes [71].

### 3.2. 16S rRNA Gm1145

In the large mitoribosomal subunit two universally conserved methylated nucleotides could be found, Gm1145 and Um1369; both are ribose 2′-*O*-methylated and both are located in the loops interacting with CCA ends of tRNA molecules bound to the P and A site respectively (Figure 2). 16S rRNA nucleotide Gm1145 is located in the P-loop of ribosome named due to the direct basepairing of the adjacent residues with the penultimate CC nucleotides of tRNA in the P-site [72]. In bacteria equivalent nucleotide Gm2251 is modified by RlmB. However, despite a universal conservation and location in the functionally important region, lack of G2251 modification in *E. coli* due to a deletion of *rlmB* gene doesn’t result in any observable difference from wild type cells [73] except for the mild reduction of ectopic expression of foreign gene introduced on a plasmid [58].

In the cytosolic ribosomes of both yeast and mammals equivalent nucleotides Gm2619 and Gm4196 are also ribose methylated [3]. However, to introduce these modifications, eukaryotes rely of the snoRNA guided mechanism [74,75].

Unlike cytosolic ribosomes that are mainly modified by snoRNA guided mechanism, mitochondrial rRNAs are methylated exclusively by specific proteins. Yeast Pet56 protein is homologous to RlmB. Deletion of *pet56* promotor results in a lack of methylation of nucleotide G2270, which is equivalent to Gm1145 nucleotide of human mitochondrial 16S rRNA [76]. Inactivation of *pet56* and its reduced expression leads to impaired growth on non-fermentable carbon sources at 30 °C, impaired oxygen consumption at 18 °C and decreased amount of fully assembled LSU [76,77]. Most likely Pet56 is active at early steps of LSU assembly as it can methylate deproteinized 16S rRNA in vitro [76].

RlmB ortholog in mammals is MRM1. Knockdown of its gene was shown to result in a decrease of the 16S rRNA nucleotide G1145 ribose methylation [78]. Most likely the protein acts co-transcriptionally because it colocalizes with mt-DNA and migrates with nucleoid in sucrose gradients [79]. Methylation of G1145 by MRM1 was found to depend on the activity of MTG2/GTPBP5, which is mainly associated with the function of MRM2 (see below) [80].

### 3.3. 16S rRNA Um1369

Methylation of the 16S rRNA nucleotide Um1369, equivalent to *E. coli* 23S rRNA nucleotide Um2552, is almost universally conserved [3] with the exception of some gram-positive bacteria [81]. This nucleotide is located in the A-loop, next to the nucleotide G2553 (*E. coli* numbering), which forms a base pair with the nucleotide C75 of A-site bound tRNA [82,83]. In gram negative bacteria, nucleotide Um2552 is methylated by RlmE (FtsJ) [84], which is co-expressed with a number of heat shock genes. Inactivation of bacterial *rlmE* gene results in growth retardation [85], which is most severe among all knockouts of rRNA methyltransferases in *E. coli* [58]. Without RlmE, the amount of 70S ribosomes decreases, while the 45S precursor of the large ribosomal subunit accumulates. This protein can methylate in vitro both 45S assembly intermediate and 50S subunits isolated from knockout strain, leading to a suggestion that it naturally acts during late steps of assembly [84,85,86]. Mutation U2552C of the bacterial 23S rRNA also results in the 45S assembly intermediate accumulation, while RlmE binding is not affected, suggesting that mere RlmE binding is not sufficient to assist ribosome construction.

Overexpression of two GTPases, ObgE (YhbZ) and EngA, complement phenotypic manifestations of *rlmE* gene knockout, but do not restore U2552 methylation [87]. Deletion of *engA* gene results in similar consequences as inactivation of *rlmE*, namely LSU precursor accumulation and diminished LSU stability at low Mg^2+^ concentration [88]. ObgE prevents association of LSU with SSU until the completion of assembly or at stress conditions [89] and is needed for sister genomes segregation during cell division [90].

Yeast Mrm2 is homologous to RlmE and is responsible for 2′-O methylation of a mt-rRNA [91]. Cells devoid of the functional protein have phenotype characteristic for mitochondrial disfunction, such as inability to grow on non-fermentable carbon source at elevated temperature (37 °C), while passaging of knockout strain in a medium with glucose causes inability to grow on non-fermentable carbon source even at optimal temperature (30 °C). Overexpression of Mtg2, which is homologous to E. coli ObgE, can partially suppress this phenotype and prevent a loss of mitochondrial DNA [92]. As its bacterial homologue, Mrm2 is likely to methylate rRNA on the late stages of assembly; its substrate being LSU, but not deproteinized rRNA [91].

In mammals, nucleotide U1369 of the 16S mt-rRNA is methylated by MRM2, homologue of RlmE [78,93]. Firstly, it was erroneously considered as a nuclear protein [94] due to unsuitable placement of tags on the N-terminus, interfering with proper localization. Subsequently it was shown that the protein is localized exclusively in mitochondria and migrated with LSU and monosome in sucrose gradients [79,93]. *MRM2* knockdown results in a decrease of LSU amount, likely due to defects in the 39S subunit assembly or stability. As a consequence such cells have impaired mitochondrial translation, decreased activity of respiratory chain and decreased growth rate in medium with galactose [93]. Functional interaction of bacterial RlmE and ObgE is conserved in mammalian mitochondria. Their homologs, MRM2 and MTG2/GTPBP5 directly interacts and simultaneously bind LSU assembly intermediate [80]. Inactivation of MTG2 gene results in an accumulation of the large ribosomal subunit assembly intermediate devoid of bL36m while containing a number of assembly factors, such as MTG1, GTPBP10, MALSU1 and MTERF4, and substoichiometric methylation of U1369, indicating a functional interaction [80].

Mutation in the human *MRM2* gene causes MELAS (Mitochondrial encephalomyopathy, lactic acidosis, and stroke-like episodes), which demonstrate the importance of mt-rRNA methylation for proper mitochondria function [95].

## 4. Common Modified Nucleotides in Mitochondrial and Bacterial rRNA

Two methylated nucleotides of the 12S mt-rRNA helix 44 and a single pseudouridine in the peptidyltransferase region of the 16S mt-rRNA are similar to their bacterial counterparts.

### 4.1. 12S rRNA m^5^C841

Helix 44 of *E. coli* 16S rRNA contains a number of modified nucleotides [3,4]. Among them is m^5^C1407 whose modification is a function of RsmF methyltransferase [96]. Inactivation of *rsmF* gene lead to moderate retardation of bacterial growth in a rich medium, which is exacerbated in a minimal medium. Also, in growth competition experiments, populations of knockout cells decline from 50% to 1% after 40 generations. Most likely RsmF is active at late stage of SSU assembly because in vitro it can methylate 30S subunit but not naked 16S rRNA [96]. Remarkably, inactivation of *rsmF* gene leads to an increased ectopic expression of exogenous gene introduced on a plasmid [58] suggesting a role for this protein in control of gene expression. While in *E. coli* RsmF methyltransferase modifies only a single 16S rRNA nucleotide, in *T. thermophilus* its homologue modifies three nucleotides, equivalent to the *E. coli* 16S rRNA residues C1400, C1404 and C1407. Nucleotides C1400 and C1404 could be methylated in vitro using deproteinized rRNA as a substrate, while C1407 could only be methylated in a context of 30S subunit. At normal temperature for *T. thermophilus* (70 °C) *rsmF* inactivation don’t lead to any notable phenotype but at low temperature (60 °C) knockout strain grows slightly slower [97].

Nucleotide C844 of human mitochondrial 12S rRNA, which is equivalent to *E. coli* m^5^C1407, is unmodified. However, a nucleotide C841, corresponding to m^5^C1404 in *T. thermophilus* 16S rRNA, is C5-methylated by NSUN4 [98]. NSUN4 forms a complex with MTERF4 protein [99,100] which belongs to a family of mitochondrial transcription termination factors [101]. Surprisingly, MTERF4·NSUN4 complex interacts mostly with the large mitoribosomal subunit [102], while the methyltransferase activity of NSUN4 is required for the small subunit modification. This complex turned out to be important for mitoribosome assembly [100]. *Mterf4* inactivation led to embryonic lethality, while tissue specific inactivation resulted in mitochondrial disfunction phenotype: elevated transcription level of mitochondrial DNA, increased mitochondrial mass and impaired mitochondrial translation. Mitochondria with inactivated MTERF4 accumulate free ribosomal subunits, which are unable to associate to form the 55S mitoribosomes [100]. The stage of the mitochondrial large ribosomal subunit assembly which requires a binding of MTERF4·NSUN4 complex was suggested on the basis of GTPBP5/MTG2 pulldown experiments and an analysis of a composition of assembly intermediates after GTPBP5/MTG2, MRM2 and mL36 gene knockdowns [80]. As suggested, it should bind after MALSU1·L0R8F8·mt-ACP module whose structure was visualized by cryo-EM [31], prior to the final stage of GTPBP5/MTG2 binding, U1369 methylation by MRM2 and association of mL36, finalizing LSU assembly.

An open question is whether MTERF4·NSUN4 complex function in the large mitoribosome subunit assembly is coordinated with the methyltransferase activity of NSUN4 towards the small mitoribosomal subunit. NSUN4 ability to modify the 12S mitochondrial rRNA do not require its association with MTERF4 [98], since *Mterf4* gene inactivation has not abolished m^5^C841 formation. It is also likely that NSUN4 methylates 12S rRNA after the formation of m^4^C839 by METTL15, since inactivation of the latter inhibits NSUN4 ability to modify 12S rRNA [10,12,13]. Inactivation of a mitochondrial methyltransferase METTL17, whose target is yet unknown, also leads to a decrease in the efficiency of C841 methylation [103]. At the same time, methylation of C841 by NSUN4 does not influence an efficiency of m_2_^6^A936 and m_2_^6^A937 methylation by TFB1M [98].

*Nsun4* gene inactivation in mice led to the same consequences as inactivation of *Mterf4*. Whole body knockout leads to the embryonic lethality, while tissue specific knockout in the heart leads to impaired mitochondrial translation, elevated level of transcription and mitochondrial mass increase, likely due to some compensatory mechanism. The reason of all of that is likely the same—accumulation of free ribosomal subunits and decreased amount of monosome. How and whether modification of the small subunit by NSUN4 is coordinated with the large ribosomal assembly facilitated by NSUN4·MTERF4 complex is unknown. According to the suggested model [98] NSUN4 modifies the small subunit independently, while NSUN4·MTERF4 complex binds an intermediate of the large ribosomal subunit assembly and following its completion, mediate subunit association.

### 4.2. 12S rRNA m^4^C839

In *E. coli* 16S rRNA nucleotide m^4^Cm1402 is located in the helix 44 approaching mRNA in the P-site. Its nucleobase methylation is done by RsmH, while ribose is methylated by RsmI [104]. Lack of either protein increases the doubling time of bacteria while in double knockout strain this effect is more pronounced. Both of the proteins could modify, albeit inefficiently, SSU isolated from the corresponding knockout strain in vitro, but neither 70S ribosomes nor deproteinized rRNA could be methylated. This indicates that RsmH and RsmI target is likely to be a late assembly intermediate, close to the 30S subunit in its composition. Ribosomes without C1402 base methylation are able to start translation from non-canonical start codons more effectively when the wild type ones, while ribosomes without the ribose modification were shown to erroneously recognize UGA as a sense codon more frequently [104].

In mammalian mitochondria, this nucleotide is methylated by a homologue of bacterial RsmH, METTL15 [10,12,13]. The phenotype of *METTL15* gene inactivation is a matter of some discrepancies. Studies performed on HAP1, human haploid adherent cell line, demonstrated that *METTL15* inactivation resulted in impaired mitochondrial translation, decreased activity of ETC complexes and decreased level of SSU and LSU association [10,12]. In the case of *Mettl15* gene inactivation in murine suspension cell line NS0, no decrease in mitochondrial translation or ETC complexes activity were observed [13]. However, the amount of free LSU and SSU was shown to slightly increase in the case of overexpression of the catalytically dead METTL15 protein.

METTL15 is involved in the assembly of the mitochondrial small ribosomal subunit. Immunoprecipitation of METTL15 allowed our group to identify a composition of its substrate complex [13]. While it contains the 12S rRNA and almost entire set of the small subunit mitochondrial r-proteins, it lacks mS37, mS38 and mS39 proteins. The protein mS39 binds the small subunit from the cytoplasmic side and facilitate a recruitment of mRNAs [39,40]. Proteins mS37 and mS38 are located in the platform region of the small subunit [96] and also contribute to the interaction with mRNAs [37,38]. The latter protein is nearest to the modification site. Additionally to the mitochondrial proteins (with above mentioned exceptions) the complex of METTL15 with the small ribosomal subunit assembly intermediate contained RBFA [13]. This protein was previously demonstrated to function as an assembly factor for the small mitoribosomal subunit [105], whose absence, albeit almost neutral for the efficiency of mitochondrial translation, lead to substoichiometric 12S rRNA methylation by TFB1M. In bacteria the function of RbfA homologue is better studied. It is known to participate in the very late stages of the small ribosomal subunit assembly preventing recruitment of the 30S into translation until the assembly is completed [106]. Structure of the 30S·RbfA complex investigated by a cryo-EM, demonstrated that the protein binds next to the 16S rRNA helices 44 and 45 [107], roughly at a place occupied by mS38 in the mitochondrial ribosomes. Moreover, RbfA induces substantial displacement of these helices and disturbs their conformation in the region approximately corresponding to the location of the 16S rRNA nucleotide C1402. It might be hypothesized that a complex of bacterial 30S subunit precursor bound to RbfA might be a substrate of RsmH, similar to that for the mitochondrial RBFA and METTL15 proteins. In addition to RBFA, methyltransferase TFB1M which is engaged to the late steps of the SSU assembly and whose activity is stimulated by RBFA [105] was also found coprecipitating with METTL15 [13]. Another mitochondrial rRNA methyltransferase, NSUN4, was found to act inefficiently upon inactivation of METTL15 gene [10,12,13], which is discussed in a previous section. In turn, activity of METTL15 was found to be reduced upon inactivation of the mitochondrial methyltransferase METTL17 [103].

Lack of active METTL15 leads to a substoichiometric inclusion of mS12, mS15, mS17 and mS38 proteins into the human mitochondrial small ribosomal subunit in HAP1 cells [10], albeit this was not observed for the murine NS0 cells [13]. Additionally we observed RBFA redistribution between the 55S ribosome and SSU so that upon inactivation of METTL15 gene RBFA might be found not only in the fraction of small ribosomal subunits, but also in that of 55S ribosomes [13], which is likely to represent suboptimal function of an assembly quality control mechanism.

While RsmH/METTL15 function in bacteria and mitochondria to methylate N4 amino group of the nucleotide C1402/C839, the corresponding nucleotide of the archaeal or eukaryotic cytosolic ribosomes is either unmodified or modified at the ribose hydroxyl [3]. This difference might be explained by a mutually exclusive presence of either m^4^C introduced by RsmH [104]/METTL15 [10,12,13] or m^6^A (m^6^A1832 in human cytosolic 18S rRNA numbering) introduced by METTL5 [108]. The latter modification might be found in the cytosolic ribosomes of eukaryotes [109] or in archaeal ribosomes [110]. These two nucleotides are forming a non-canonical basepair in all organisms, so that a single methyl group is placed in roughly equivalent position while attached to either one of these nucleotides. Such mutually exclusive modifications are common in ribosomes [3]. It remains unclear as to whether the methylation of m^4^C1402/C839 and m^6^A1832 have similar functional roles or these modifications are specifically adapted for bacterial-type and archaeal-type ribosomes.

### 4.3. 16S rRNA Ψ 1397

16S rRNA Ψ1397 is the only pseudouridine in human mitochondrial ribosome [7]. It is formed by RPUSD4, an enzyme also responsible for the modification of mt-tRNA^Phe^ [111,112]. Nucleotides in yeast mitochondrial [113] and bacterial ribosomes [114,115] equivalent to Ψ1397 are pseudouridinylated. *E. coli* RluC pseudouridine synthase is responsible for modification of the 23S rRNA nucleotides Ψ955, Ψ2504 and Ψ2580; the latter being equivalent to the mitochondrial 16S rRNA Ψ1397 residue. Mitochondrial 16S rRNA unlike bacterial 23S rRNA do not have an equivalent to U955, while an equivalent to U2506 is present, although unmodified. It seems likely, but could not be unequivocally stated, that RluC and RPUSD4 are orthologues; RPUSD4 bears a significant similarity with a number of bacterial pseudouridine synthases, such as RluA [116], which have an ability, like RPUSD4, to modify tRNA [117]. Inactivation of *rluC* gene in bacteria and the corresponding PUS5 in yeast has very mild phenotype [113,114].

Formation of 16S rRNA Ψ1397 modification in mammalian mitochondria seems more important. RPUSD4 gene, responsible for formation of this modification, was found by a genome-wide CRISPR/Cas9 gene inactivation screen as essential for oxidative phosphorylation [118]. *RPUSD4* knockdown results in a decrease of the 16S rRNA, LSU and monosome amounts leading to impaired mitochondrial translation and decreased activity of ETC complexes [111]. RPUSD4 mediated pseudouridinylation of mt-tRNA^Phe^ seems less important than modification of the 16S rRNA, since this modification influences neither amount of the tRNA^Phe^ nor its aminoacylation level [112].

## 5. 16S rRNA Nucleotide Gm1370 Is Methylated in Mitochondrial and Cytosolic Ribosomes

While several nucleotides of mitochondrial rRNAs are modified in a way similar to that in bacteria, there is a remarkable exception. Nucleotide Gm1370 of the ribosomal A-loop is modified in mitochondrial [6] and cytosolic [119] ribosomes of eukaryotes, as well as in archaea [120], but not in gram-negative bacteria. Interestingly, the presence of Gm nucleotide in the A-loop was documented in some gram-positive bacterial species [81], although the enzyme responsible for this modification has not been identified. Mitochondria are likely to acquire this rRNA modification from archaea that was a predecessor of eukaryotic cell that engulfed bacterial predecessor of mitochondria. A bacterial origin of this modification is less likely, since gram-negative proteobacterial predecessor of mitochondria possibly did not have it.

Although in gram-negative bacteria equivalent 23S rRNA nucleotide G2553 is not methylated, it is functionally very important because it forms a basepair with nucleotide C75 of the A-site bound tRNA [82,83]. Yeast cytosolic ribosomes are modified at equivalent 25S rRNA nucleotide Gm2922 by a specialized protein Spb1 acting late in the ribosome assembly process [121]. Yeast strain with inactive Spb1 mutant is cold-sensitive and has ribosome subunit association defect. It is of interest to note that neighboring nucleotide Um2921 of yeast cytosolic 25S rRNA, although universally conserved, is modified by snoRNA guided mechanism, while for the modification of Gm2922 a specialized protein methyltransferase was used. This peculiarity may lead to the suggestion that Spb1 methyltransferase has a separate role in ribosome assembly, which goes beyond a mere introduction of the methyl group.

Ribose methylation of G1370 in human 16S mt-rRNA is done by MRM3 [78,93]. It is localized in mitochondria and on sucrose gradients co-migrates with nucleoid and LSU [79,93]. Not only LSU proteins, but also RNA-chaperones (p32/C1QBP, LRPPRC, PTCD3 and GRSF1), assembly factors MTERF3 and DDX28 and 16S rRNA pseudouridine synthase RPUSD4 co-precipitate with MRM3 [79]. In the case of MRM3 gene knockdown LSU assembly intermediate is accumulated [93] and, as results of it, mitochondrial translation is impaired [79,93], ETC complexes activity is decreased and cells growth is slowed on either glucose or galactose containing medium [93]. To summarize it, the MRM3 target is assembly intermediate of 39S subunit and the presence or activity of the protein are important for proper ribosome assembly.

## 6. rRNA Nucleotides Methylated Exclusively in the Mitochondrial Ribosomes

Apart from the ribosomal RNA modification machinery acquired from the bacterial and archaeal predecessors of eukaryotic cells, a couple of rRNA methyltransferases evolved to function exclusively in the mitochondria to make modification which could not be found in any other ribosomal RNA outside this organelle.

### 6.1. m^1^A947 16S rRNA

In the 3D structure of the mitochondrial ribosome 16S rRNA nucleotide m^1^A947 is located close to the methylated nucleotides of the A-loop (Figure 1). Modification of this nucleotide is specific for mammalian mitochondria and it is the only methylation of a heterocyclic base in the 16S mt-rRNA.

This nucleotide is modified by the dual-specific enzyme TRMT61B, the other target of which is mt-tRNA. An ability to modify nucleotide A58 of mitochondrial tRNA was discovered first, driven by a homology of TRMT61B to a family of tRNA methyltransferases responsible for making m^1^A58 nucleotide modification [122]. In bacteria, a homologue of this enzyme, TrmI, acts as a homotetramer [123,124], while yeast cytoplasmic tRNAs are modified by a heterotetramer composed of Trm6·Trm61 dimers [125] which had likely been evolved due to gene duplication. Human orthologues of these proteins, TRMT6 and TRMT61A, are responsible for the cytoplasmic tRNA modification [126]. Another gene duplication event which is likely to happen in the evolution of vertebrates resulted in the formation of a paralogous TRMT61B gene.

Nucleotide m^1^A58 of tRNA forms reverse Hoogsteen base pair with m^5^U54 in T-loop [127]. It is considered that A58 methylation introduces positive charge to the base and stabilize tertiary tRNA structure [128].

Modified nucleotide m^1^A947 in the mitochondrial 16S rRNA was serendipitously discovered by difference in sequences of DNA and RNA (RDD, RNA-DNA difference) [129]. In the sequence of mt-DNA only adenine was observed at this position, while in RNA sequence reads adenine, thymine or guanine could be found due to erroneous nucleotide incorporation into cDNA opposite to the m^1^A by a reverse transcriptase. Inactivation of the TRMT61B gene resulted in the loss of this 16S rRNA modification supporting the idea that this enzyme acquired a new target, mitochondrial rRNA, in addition to the mitochondrial tRNA molecules.

It is hypothesized that m^1^A947 facilitates helix H71 interaction with H64 and H92 in 39S subunit. However, no phenotype of the TRMT61B gene knockdown was found. Corroborating the moderate influence of TRMT61B on organism fitness is the fact that some species, such as *M. musculus* and *O. anatinus*, don’t have a functional *TRMT2B* gene which is accompanied by a substitution of the corresponding target nucleotide in the 16S rRNA. In majority of bacterial ribosomes the nucleotide corresponding to the mitochondrial 16S rRNA nucleotide m^1^A947 is G, while in eukaryotic cytosolic ribosome this position is occupied by T. Artificial substitution of the equivalent nucleotide in *E. coli* ribosomes demonstrated that while G to T substitution has virtually no consequences, G to (unmodifed) A substitution decreased the growth rate and impaired in vitro and in vivo translation efficiencies [129]. It is thus possible that m^1^A947 modification might prevent formation of some unproductive interactions that might be formed by the adenine at this position.

### 6.2. 12S rRNA m^5^U429

Modified 12S rRNA nucleotide m^5^U429 is unique for vertebrate mitochondria; equivalent nucleotides in other types of ribosomes are not modified. It was recently demonstrated that U429 is methylated by protein TRMT2B [9,11], a dual specificity enzyme which is also capable to form m^5^U54 residue in the T-loop of a number of mitochondrial tRNAs. Although equivalent nucleotides in other ribosomes are not modified, bacteria and yeast have homologues tRNA-specific methyltransferases TrmA [130] and Trm2 [131]. Reconstruction of the phylogenetic tree of C5 uridine RNA methyltransferases allows us to suggest that this type of enzyme is related to bacterial 23S specific RNA methyltransferases RlmD, RlmC and RlmCD whose evolution in bacteria [132,133] is outside a scope of this review (see [3] for a review). While invertebrates possess a single representative of this family, Trm2, responsible for the cytosolic tRNA modification [131], vertebrate genomes encode its two paralogues, TRMT2A and TRMT2B. TRMT2A is capable to modify cytosolic tRNA [134], while TRMT2B became specialized not only in mitochondrial tRNA modification, but also in modification of the 12S rRNA. This evolutionary history is reminiscent to the emergence of the dual specificity methyltransferase TRMT61B. However, unlike TRMT61B gene inactivation, which is phenotypically silent, TRMT2B gene inactivation resulted in a moderate decrease in the activity of ETC complexes containing mitochondrially encoded subunits, while the efficiency of mitochondrial translation was apparently unaffected [9,11].

**Figure 3:**
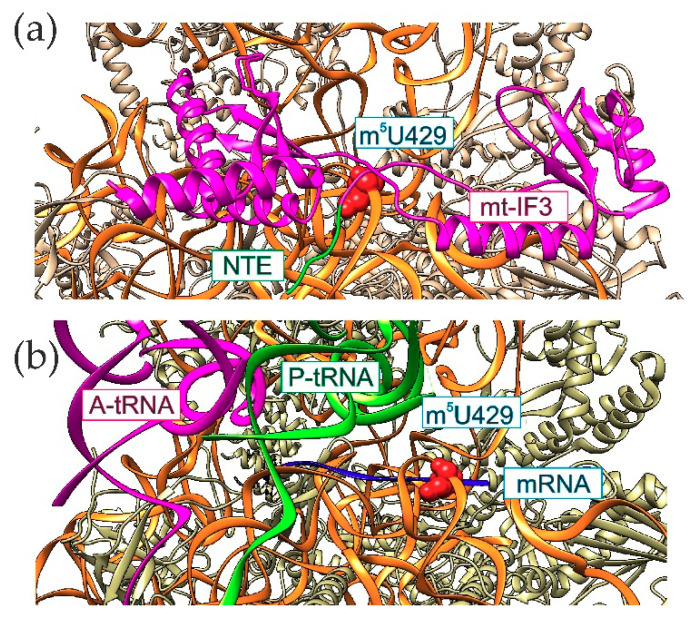
Location of the methylated nucleotide m^5^U429 of the 12S rRNA (red spacefilled object) relative to the ligands of the small subunit of mitochondrial ribosome. (**a**) tertiary structure of mt-IF3 complex with the 28S subunit [135]. mt-IF3 is shown as pink ribbon, while its mitochondria-specific N-terminal extension is shown as green ribbon. (**b**) tertiary structure of the mammalian mitochondrial ribosome complex with mRNA and tRNAs [69]. Color scheme is the same as for the Figure 2. UCSF Chimera [36] was used to create this illustration.

Location of the modified 12S rRNA m^5^U429 in a conserved functionally important region of the mitochondrial ribosomal subunit (Figure 1b) suggest that it may have specific function in mitochondrial protein synthesis. In line with this hypothesis is a direct contact of m^5^U429 with the mitochondrially specific extension of the translation initiation factor mt-IF3 [135] (Figure 3a) and mRNA at the position 5′ to the P-site [69] (Figure 3b). Both might be relevant to the peculiarities of mitochondrial translation initiation. However, without more experimentation the role for the 12S rRNA m^5^U429, modification remains elusive.

## 7. Conclusions

**Figure 4:**
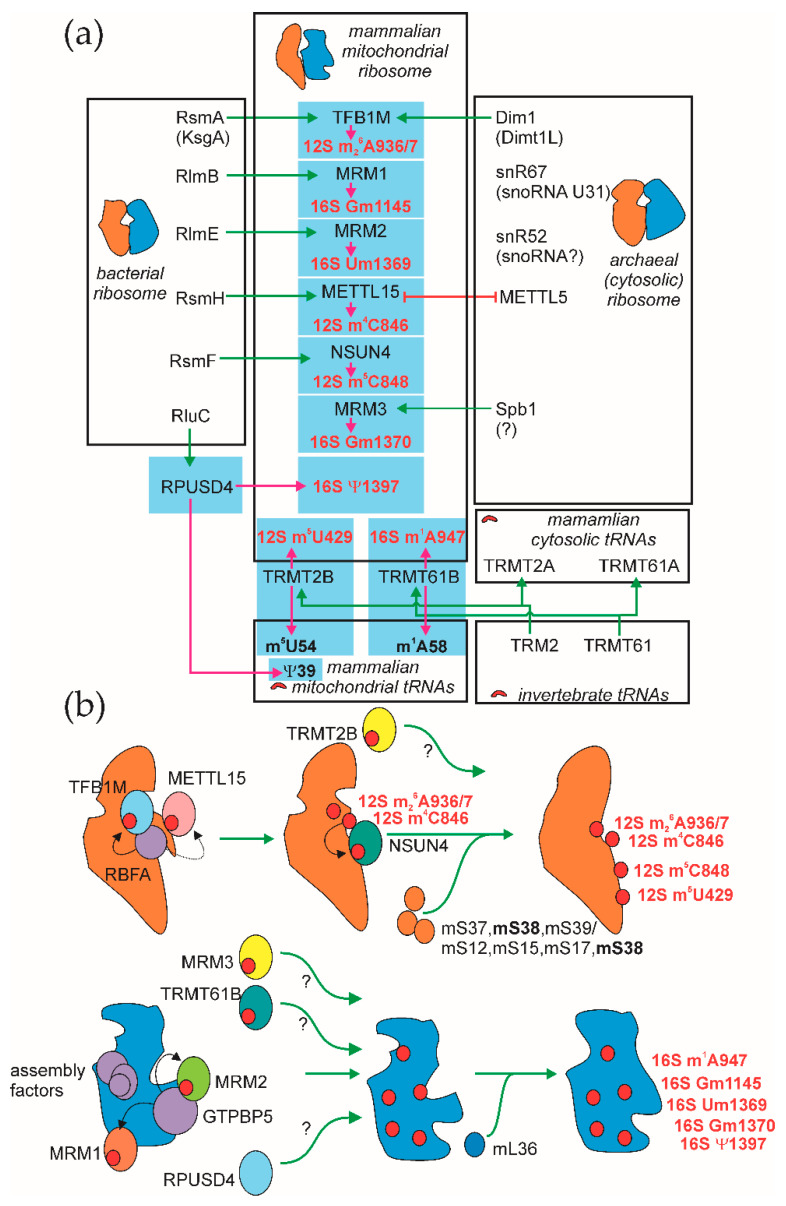
Mitochondrial rRNA modification in the context of evolution and mitochondrial ribosome assembly. (**a**) Likely evolutionary origin of enzymes responsible for mammalian mitochondrial rRNA modification. Boxed are the systems for the methylation of mammalian mitochondrial rRNA (center), related part of the bacterial (left) and archaeal/eukaryotic cytosolic (right) rRNA modification systems. Related systems of tRNA methylation are shown on the lower part of the panel. Pink arrows demonstrate enzymatic activities. Green arrows demonstrate possible evolutionary origin. Mutually exclusive presence of METTL15 and METTL5 is shown by a red blunt-ended arrow. (**b**) A model for the involvement of mitochondrial rRNA modification enzymes in the assembly of the small (upper part) and the large (lower part) mitochondrial ribosome subunit assembly. Orange objects corresponds to the small subunit components, while blue ones to that of the large subunit. Red circles correspond to methyl groups transferred by rRNA methyltransferases and pseudouridine formed by RPUSD4. Black arrows correspond to the stimulatory interactions; dotted arrow to the putative one.

While the eukaryotic cell is likely to be a product of symbiosis of primordial archaea and bacteria, mitochondrial rRNA modification machinery originated from both of these sources (Figure 4a). Bacterial-type modifications are likely to reflect a more substantial similarity of mitoribosomes, as well as a translation apparatus in general to bacterial ones. On the other hand, at least one modification, of the 16S rRNA nucleotide Gm1370, is likely to be acquired from the archaea/cytosolic ribosome modification apparatus. Two modifications of the vertebrate mitoribosomes have not been inherited from other ribosomal modification systems, 12S rRNA m^5^U429 and 16S rRNA m^1^A947. Both of these modifications appeared as a result of duplications of genes coding for tRNA-specific RNA metyltransferases and both enzymes thus formed, TRMT2B and TRMT61B, have dual specificity (Figure 4a). Due to this fact, it remains an open question as to whether modification of rRNA by these proteins is a mere byproduct of tRNA modification, or does it have a special function? For another dual specificity mitochondrial rRNA/tRNA modification enzyme, RPUSD4 pseudouridine synthetase, the primary function is likely to be modification of rRNA.

While a detailed map of the mitochondrial ribosome assembly is only emerging [80,136,137,138,139], few snapshots of this complex process involving rRNA methyltransferases might be drawn (Figure 4b). Small subunit assembly factor RBFA (Figure 4b, upper panel) is known to positively influence activity of TFB1M and coprecipitate with METTL15. Activity of METTL15 is needed for efficient functioning of NSUN4, placing the latter downstream. While there are discrepancies on the particular set of r-proteins which joins 28S subunit after the methylation of its precursor by METTL15; mS38, the protein closest to the modification site, was included into this list by all researchers. Nothing is known about the timing of TRMT2B action.

During construction of the large 39S subunit, an intermediate containing a set of assembly factors binds GTPBP5, interacting with and stimulating the activity of MRM2 (Figure 4b, lower panel). It is known that MRM1 activity is also stimulated by this factor. Association of the ribosomal protein mL36 happens downstream. The timing of RPUSD4, MRM3 and TRMT61B functioning is yet unknown.

While the list of enzymes responsible for the modification of mammalian mitochondrial rRNA is completed, further studies are needed to illuminate all details of the mitochondrial ribosome assembly and maturation map. The same is true for the investigation of the roles modified nucleotides play in the mitochondrial translation process.

## Figures and Tables

**Table 1:** Enzymes responsible for the modification of mammalian mitochondrial rRNA (human numbering).

	Enzyme, Responsible for Mitochondrial rRNA Modification	Orthologous Bacterial Protein	Orthologous Protein Responsible for Archaeal or Cytosolic rRNA Modification
12S rRNA			
m^5^U429	TRMT2B	no	no
m^4^C839	METTL15	RsmH	no
m^5^C841	NSUN4	RsmF ^1^	no
m_2_^6^A936/7	TFB1M	RsmA/KsgA	DIMT1L (yeast Dim1)
16S rRNA			
m^1^A947	TRMT61B	no	no
Gm1145	MRM1	RlmB	U31 (yeast snR67) snoRNP
Um1369	MRM2	RlmE	Unknown ^2^ (yeast snR52)
Gm1370	MRM3	no	Unknown ^2^ (yeast Spb1)
Ψ1397	RPUSD4	RluC ^3^	no

^1^*E. coli* RsmF modifies the nucleotide m^5^C1407 of the 16S rRNA, which is proximal to C1404, equivalent to m^5^C841 of the mitochondrial 12S rRNA, while *Thermus thermophilus* RsmF modifies 16S rRNA residues m^5^C1400, m^5^C1404 and m^5^C1407 (*E. coli* numbering). ^2^ There are no direct experimental data on the proteins and/or snoRNA involved in these modifications in human rRNA. ^3^ *E. coli* RluC forms 23S rRNA pseudouridines Ψ955, Ψ2504 and Ψ2580. The latter is equivalent to Ψ1397 of the mitochondrial 16S rRNA.
